# Starting from scratch: Ziermans et al. commentary

**DOI:** 10.1016/j.scog.2025.100414

**Published:** 2025-12-10

**Authors:** David Dodell-Feder, Steven M. Silverstein

**Affiliations:** a Department of Psychology, University of Rochester, Rochester, NY, 14627, USA; b Department of Neuroscience, University of Rochester Medical Center, Rochester, NY, 14642, USA; c Department of Psychiatry, University of Rochester Medical Center, Rochester, NY, 14642, USA; d Department of Ophthalmology, University of Rochester Medical Center, Rochester, NY, 14642, USA

If you have ever made pancakes, you know that the first one inevitably comes out poorly: an unpleasant mélange of pale yellows and dark browns, burnt on the edges, and yet undercooked in the center. In fact, the first several are often bad. Edible, yes, but you need to dress them up considerably, dousing them with syrup and butter, or cutting away the burnt and undercooked bits, leaving an unsatisfying morsel with minimal similarities in flavor, texture, and appearance to a pancake. It is usually better to walk away—make your sacrifice to the griddle Gods and start from scratch. Daunting, especially if you are in the throes of a morning routine, but worthwhile because pancakes are important.

If you squint (considerably), you might see parallels between pancakes and social cognitive assessment in clinical samples. As incisively described in Ziermans et al. (2026), a global survey clearly shows that our available tools for assessing social cognition are falling short. These measures are our field's first several pancakes—usable, yes, but with considerable drawbacks including a lack of normative data, limited cross-cultural utility, limited ecological validity, and problematic psychometric properties, among other issues. Equally as disheartening, Ziermans et al. find that many of us (the authors included) keep using the same measures that we know are most problematic (e.g., the Reading the Mind in the Eyes Task).

Ziermans et al.'s call to action is a compelling one. We wholeheartedly agree that “validation of core measures and systematically adapting them across cultures” is a critical initiative, the likes of which has borne some of the most important data to date regarding social cognitive assessment in schizophrenia (Pinkham et al., 2018). But, as inveterate pancake cooks, we cannot help but wonder if those efforts might fall short of our expectations, and simply demonstrate that many of our core measures lack validity according to most metrics (e.g., Higgins et al., 2024) and/or defy cultural adaptation (e.g., Wu and Keysar, 2007). The financial and potential opportunity costs of such an effort would be unfortunate, as would be the empirical realization of a worst-case scenario.

As such, we wonder whether it might be worthwhile prioritizing new measure development; that is, starting from scratch. There has been much exceptional work on social cognitive theory in the past several years, deconstructing social cognitive processes into its measurable, constituent parts (Conway et al., 2019; Quesque and Rossetti, 2020; Schaafsma et al., 2015; Tamir et al., 2016), which could facilitate the development of measures grounded in empirically-supported frameworks that may better capture and distinguish social information processes. These efforts may too present opportunities for useful riffs on the typical outcome assessed, which is accuracy. Accuracy can be fuzzy without a ground truth, which many tasks do not assess performance against (sometimes because the ground truth is unavailable; c.f., Zaki et al., 2008). While accuracy in social attribution is indisputably important, there are likely other critical, yet understudied inferential processes or features of social cognition to assess, like confidence in (Jones et al., 2020), introspective accuracy for (Springfield and Pinkham, 2020), and decisiveness in social attributions, the size of one's hypothesis space for another person's mental states and the way in which that hypothesis space gets pruned by the social context or target, the spontaneity of and propensity/motivation for making social attributions (Pomareda et al., 2024; Rice and Redcay, 2015), the structure, organization, and application of social knowledge (Thornton et al., 2022), and other processes that may help or prevent someone getting to an accurate inference (Eyal et al., 2018).

Developing new measures—especially ones that might address multiple obstacles described by the experts in Ziermans et al.—is a gargantuan task, which we recognize is easy to for us call for, yet hard to do. It is also an effort in need of sustained funding, trainees, and administrative support, which is difficult to come by in the current climate for many of us. That is all to say, focusing on new measure development is not the easy route—problems and obstacles abound. And, there is an argument to be made about the opportunity cost of such an investment, while ongoing intervention research, for example, is stuck using the available, and in many ways, inadequate measures. But, we think starting from scratch is worth considering as the priority, rather than spending the time and effort dressing up the measures we have at hand. Prior and existing consortiums convened to evaluate social cognition (e.g., the SIRS Research Harmonization group that authored the target article) could provide a useful framework for such an initiative. However, history suggests that developing new frameworks and recommendations does not always lead to progress. Several useful recommendations for social cognition test development for schizophrenia, and short- and long-term research agendas were recommended years ago in the MATRICS New Approaches Conference (Green et al., 2005), the NIMH Social Cognition Workgroup (Green et al., 2008), and in other contexts (Silverstein, 1997), but few of these recommendations have been followed. We therefore recommend that new test development be prioritized to reach the goals outlined in Ziermans et al. in addition to addressing a host of other long-recognized needs in this field related to: demonstration of relationships between test scores, arousal, affect, and social behavior; considerations of feedback loops between social behavior and social cognition; adequacy of carrying out multiple social cognitive operations simultaneously; clarification of cognitive and neurobiological mechanisms involved in test performance and real-world social functioning; and development of new models of cognitive and social cognitive impairment in clinical conditions (e.g., the role of predictive processing [Li and Li, 2025; Tamir and Thornton, 2018] and context processing [Phillips et al., 2015; Schenkel et al., 2005] failures in influencing both sets of functions).

We are eager to see the directions that researchers in the field take, and are grateful for the efforts of Ziermans et al.'s and similar other efforts (Corbera et al., 2025; Pinkham et al., 2018) for starting and continuing this important conversation.

## CRediT authorship contribution statement

**David Dodell-Feder:** Conceptualization, Writing – original draft. **Steven M. Silverstein:** Conceptualization, Writing – review & editing.

## Funding sources

We received no funding support for this work.

## Declaration of competing interest

We have no conflicts of interest to report in relation to this commentary.
